# Streamlining Cutaneous Melanomas in Young Women of the Belgian Mosan Region

**DOI:** 10.1155/2014/320767

**Published:** 2014-02-25

**Authors:** Trinh Hermanns-Lê, Sébastien Piérard

**Affiliations:** ^1^Department of Dermatopathology, Unilab Lg, University Hospital of Liège, 4000 Liège, Belgium; ^2^Dermatology Unit, Diagnostic Centre, 4800 Verviers, Belgium; ^3^INTELSIG Laboratory, Montefiore Institute, University of Liège, 4000 Liège, Belgium

## Abstract

Sporadic cutaneous melanoma (SCM) has shown a dramatic increase in incidence in Caucasian populations over the past few decades. A particular epidemiological increase was reported in women during their childbearing age. In the Belgian Mosan region, a progressive unremitting increase in SCM incidence was noticed in young women for the past 35 years. The vast majority of these SCMs were of the superficial type without any obvious relationship with a large number of melanocytic nevi or with signs of frequent and intense sunlight exposures as disclosed by the extent in the mosaic subclinical melanoderma. A series of investigations pointed to a possible relationship linking the development of some SCM to the women hormonal status including the effect of hormonal disruptors. These aspects remain, however, unsettled and controversial. It is possible to differentiate and clearly quantify the SCM shape, size, scalloped border, and variegated pigmentation using computerized morphometry as well as fractal and multifractal methods.

## 1. Introduction

The sporadic cutaneous melanoma (SCM) incidence has risen over the past decades across various groups of Caucasian populations [1–3]. Such an increase is possibly genuine or caused, in part, by intensive population screenings in individuals of light complexion and by early detection procedures. A large number of nevi are apparently the strongest risk factor for SCM in Caucasians [4]. The numerical density in nevi is a heritable characteristic with about 60% of the variation attributed to other additional genetic effects. Fair presentations of skin and hair represent a combination of risk factors whose magnitude remains much smaller. Different risks to develop SCM are associated with the phenotypes. They include presence of photodamage such as solar elastosis and actinic keratoses [5]. Globally, repeat sun exposures are linked to an increased SCM incidence with decreasing latitude. It remains that this type of environmental exposure is difficult to quantify. In addition, such an association is in part confounded by the fact that population screening in geoclimatic regions with high intensity of sunlight exposure commonly increases the detected SCM incidence.

This review is focused on gender differences in SCM incidence according to age. Both computerized morphometry and fractal analysis will be applied to the clinical aspect of the neoplasms and to the peritumoral apparently uninvolved skin.

## 2. Gender Influence and Age-Related Melanoma Incidence

In general, cancers sensitive to female sex steroids are associated with several risk factors, such as low parity, infertility, early age at menarche, and late age at menopause [4]. As far as SCM is concerned, the neoplasm develops predominantly in white people and is under gender influence [1, 6–10]. The SCM incidence rates are particularly high in Northern Europe, North America, and Australia. By contrast, they are low among the indigenous populations of Africa, Asia, Latin America, and Southern Europe. About one-third of all SCM in women develop during their childbearing age particularly between 25 and 29 years [11, 12]. Until the age of 45 years, SCM incidence rates in women exceed those in men [2, 3]. Such a trend is levelled off later [1]. This trend suggested the possible intervention of some hormonal influences [13–19]. Investigations were previously focused on women endocrine status including the possible impact of oral contraceptives, hormone replacement therapy, age at first child, age at menarche, age at menopause, menopausal status, and administration of fertility drugs [20–23]. They reflected potential influences of exogenous and endogenous hormones. In particular, a significant increase in SCM risk was reported for late age at first birth. By contrast, women with several children appeared to be at lower risk for SCM. These epidemiological findings were apparently linked to a series of socioeconomic confounders. Some concerns originated from pregnancy-related SCM, reports on skin hyperpigmentation during oral contraception, and nevi enlargement and darkening during pregnancy [17]. However, the link between SCM occurrence and pregnancy and hormonal and reproductive factors remains controversial. By contrast, gender is an established prognostic factor in SCM with women having a better overall prognosis than men [10].

The influence of fertility drugs on SCM risk has not been extensively studied despite the common use of this group of exogenous hormones over the last decades [21] and their established effect on ovulation and endogenous hormone production. Given the increasing frequency of prescriptions of fertility drugs among infertile couples, the concern about fertility drugs increasing the SCM risk has been considered as important public health worries. The impact of hormonal disruptors has been recently evoked [24–29]. They could interfere with estrogen receptors and alter the functions of some HOX genes [29, 30].

The pattern of age-incidence rates of SCM in women resembles that of breast cancer. They are higher in women than in men especially before 45 years of age. Afterwards, differently from men, rates of increase slow down [31]. Furthermore, a higher risk of breast cancer among women with a history of SCM and excess SCM risk among breast cancer patients have been reported in several studies [32–34].

## 3. Clinical Presentation

SCM exhibits several clinical presentations [35]. In our experience, these neoplasms developed in young women are generally flat and slow growing [29]. Of note, clinically featureless SCM is possibly disclosed using computerized monitoring. Basically, the clinical screening for SCM relies on the ABCDE rules in which A denotes asymmetry, B indicates border irregularity, C denotes color variegated pattern, D stands for diameter, and E indicates the evolutionary extension in size [36]. Other features are added to the ABCDE acronym, including changes in shape, shades of color, symptoms (itch, tenderness), and surface presentation (e.g., bleeding, crusting, scaling, etc.). They allow detection of smaller and morphologically featureless SCM.

The diagnosis of SCM in its early phase of development is mandatory in order to improve the prognosis and decrease mortality. For this purpose, increased interest has been paid to cyanoacrylate skin surface strippings (CSSS) [37, 38] and dermoscopy [39, 40].

CSSS is a minimally invasive method exclusively collecting the superficial layers of the stratum corneum. In melanocytic neoplasms, melanin is present inside corneocytes and eventually in atypical melanocytes. Melanin restricted only inside corneocytes is a feature of benign neoplasms such as lentigines and melanocytic nevi. By contrast, the presence of atypical melanocytes inside the stratum corneum is strongly suggestive of SCM but also, in rare instances, of a benign melanoacanthoma [37]. Thus, CSSS proves to be sensitive and specific for distinguishing SCM from benign melanocytic tumors such as common melanocytic nevi, dysplastic nevi, or pigmented seborrheic keratoses. For research purposes, karyometry of neoplastic melanocytes is conveniently performed on CSSS [38].

Dermoscopy (surface microscopy, epiluminescence microscopy) is a simple optical method leaning on incident light magnification and pattern analysis. It allows identification of morphological aspects invisible with the naked eye. Currently, there is a suggested two-stage procedure for the diagnosis of pigmented skin lesions using dermoscopy [39, 40]. The first stage permits the differentiation of melanocytic tumors from nonmelanocytic neoplasms including seborrheic keratoses, pigmented basal cell carcinoma (BCC), and hemangioma. Once a melanocytic tumor is diagnosed, the second stage allows differentiation of SCM from benign melanocytic tumors such as lentigo simplex, typical and atypical nevi, and solar lentigo.

Conventional dermoscopy does not detect all in situ SCM [41, 42]. Several descriptors characterizing in situ and thin SCM have been proposed [43, 44]. It remains that classification into different SCM subtypes is not immediately recognizable according to the established main dermoscopic patterns [45].

## 4. Computerized Monitoring

From two-dimensional images of pigmented tumors, a series of feature operators are conveniently applied to extract texture descriptors useful for clinical diagnosis [46]. Computerized monitoring devices record clinical presentations and/or digital dermoscopy images [47]. They allow tiling on the computer screen for comparison of pigmented lesions for change over time. A number of devices using a variety of cameras have ideally to be controlled by adequate calibration. A series of semiautomatic algorithms have been designed. The attractiveness of computerized monitoring is the rationale of clinical decision making. When a melanocytic tumor remains unchanged, it is assumed to be benign, and when it has changed, excision is warranted for concern about malignancy. Undoubtedly, a preliminary diagnostic procedure avoiding unnecessary excisions is preferable. The procedures are conveniently divided into two categories corresponding to short-term and long-term monitoring, respectively, [48–50].

Short-term monitoring, usually over a 3-month period, is used to establish a clinical judgment about abnormal melanocytic tumors that do not exhibit clear SCM features [49, 51]. These lesions usually belong to two groups, namely, moderately atypical melanocytic nevi and skin melanocytomas that apparently remained unchanged over time and discretely atypical melanocytic nevi exhibiting features of change. In the short-term monitoring procedure, any variation in presentation over a 3-month period, except increased or decreased milia-like cysts or overall variation in pigmentation consistent with increased or loss of tan in the surrounding skin, requires excision of the neoplasm. The specificity of the computerized morphometry for SCM reaches about 80%, and the sensitivity is near 100%, although such estimate has not been conclusively demonstrated [46–48].

Computerized short- and long-term monitorings help identifying featureless-appearing SCM that can only be detected by high resolution morphological changes. Some of these featureless SCM are conveniently demonstrated by short-term monitoring [51, 52]. Monitoring investigations raised concerns about the diagnostic accuracy of early SCM in regular clinical setting [46].

Both short- and long-term computerized monitoring readily allows detection of some featureless SCM, but the physician efficiency is subjected to interindividual differences. Long-term monitoring allows comparison of atypical melanocytic tumors over prolonged surveillance periods. These monitored atypical tumors are not considered as possible SCM at the time of imaging. Such diagnostic procedure is generally restricted to patients who have multiple dysplastic nevi [48] or enlarging compound nevi under human growth hormone therapy [53]. About 5% of pigmented neoplasms monitored show obvious changes over a 12-month surveillance period [50]. The changes correspond to tumor enlargement, alterations in shape or color, regression, and appearance of dermoscopic features SCM.

Melanocytic nevus stability depends on the age of the patient under monitoring. Although about 15% of regular nevi show prominent changes before 20 years, only about 2% of melanocytic nevi in adults older than 40 years show similar changes [54]. Such age relationship was further reported when monitoring atypical nevi, with about 10% of them changing in patients younger than 28 years of age but in only 3% of adults older than 48 years [55]. Hence, long-term computerized monitoring is particularly efficient in adults older than 40 years [46].

## 5. Computerized Morphometry and Fractal Analysis of the Clinical Aspect

Many biologic processes are known to be heterogeneous, especially in the oncologic field. Repartition of estrogenic receptors is inhomogeneous, and the heterogeneity of these variables is deduced from multiple measures sampled on different sites of the tumor. Although such evaluation does not constitute a quantitative approach, it evokes the heterogeneity of the phenomenon.

The actual size, shape, and symmetry of pigmented lesions are possibly assessed using computerized morphometry [48, 49]. The optical image of excised SCM specimens is acquired using a video camera and image analysis following a morphometric computer program. Both the SCM area and form factor (Form AR) are measured. Form AR identifying the fine irregularities of contour ranges from 0 to 1. Values close to 1 indicate a smooth and regular outline. The area can be changed to a circle the diameter of which (D circle) is calculated.

When data do not exhibit a Gaussian distribution, the Kolmogorov-Smirnov test and the Mann-Whitney *U* test are conveniently used for evaluating the differences in distributions and medians of the values. Multivariate discriminant analysis demonstrates the ability of the analytic variables to discriminate the vast majority of the melanocytic neoplasms, particularly when two-dimensional variables are included [47].

One of the major clinical diagnostic criteria for SCM is expressed by morphometry as the combination of a large D circle value and a small Form AR. These two independent parameters are combined in a ratio to define the clinical index of atypia (Ia) corresponding to Ia = D circle (Form AR)^−1^. It is higher when the D circle is large and Form AR is small, lower than the value 1 [49].

The variegated SCM aspect is possibly analysed using fractal analysis because the fractal concept of self-similar structures is applicable to clinical pictures of some skin neoplasms [56–58]. Optical images are commonly acquired using a video camera and digitized on a matrix of 512 × 512 pixels with 256 gray levels. A texture analysis on the gray levels of the SCM images is conveniently performed by means of fractal characterization using both fast Fournier spectrum and multifractal analysis [57]. Contiguous optical fields are observed and measurement data are averaged before using specific algorithms [57]. Enhancement of local discontinuities or edges is performed using a gradient technique. The identification of structures considered as fractal is in part linked to the scaling characteristics that have to follow power laws. The goodness of fit of the linear regression in the log-log plot is essential. The actual values should lie as close as possible to a straight regression line, and the slope gives an estimate of the fractal dimension. This condition is fulfilled when the *R* ^2^ values of the regression is close to 1. Moreover, the histograms of the scattered residuals around this line should follow a normal distribution [58].

Various patterns of variegated SCM pigmentation are observed both in men and women. The interpretation of power spectrum images is generally rather difficult, except when the images have particular sizes or orientations. If the pattern is not really periodic or if it is noisy, the main frequency is hidden by a halo that is denser as the structure becomes more complex [57].

No gender-related differences have been disclosed so far using the parameter of SCM morphometry and fractal analysis.

## 6. Peritumoral Mosaic Subclinical Melanoderma

Skin photoprotection depends in part on the uppermost keratinocytes with the melanin pigment producing melanocytes present in the basal layer of keratinocytes. Melanin is synthesized in the melanocytes and helps protecting the skin from the deleterious effects of ultraviolet light (UVL) radiations by several mechanisms. Accordingly, patients with impaired production of melanin suffer from a higher incidence of skin cancers. The relationship between the total melanin content, the eumelanin : pheomelanin ratio, and the activity of the key melanogenic enzyme tyrosinase is complex. Mature melanosomes filled with melanin pigment are transported from the melanocyte cell body into the dendrites, to be transferred to the keratinocytes, where they localize to the uppermost perinuclear area.

The mosaic subclinical melanoderma (MSM), also called faint mosaic melanoderma (FMM), is a physiologic pattern of heterogeneous distribution of melanin inside the epidermis. It is influenced by repeat photoexposures since early adulthood [59, 60]. Indeed, the impact of UVL on the epidermis induces an increased production of melanin by melanocytes and its transfer to neighbour keratinocytes in each epidermal melanin unit (EMU). Such EMU activation is responsible for the presentation of MSM, particularly evident in Caucasian skin. A previous study showed that the median value of MSM extent was significantly higher in men with SCM than in women with the same neoplasm [29].

UVL exposure undoubtedly plays a role in the development of SCM, but its involvement is not as clear-cut as for other skin cancers such as squamous cell carcinoma. More risks appear to be associated with intermittent/recreational than with occupational/continuous sun exposure. However, the emphasis on “sun burning” as a cause, rather than a risk factor, is likely misplaced. Thus, for the development of SCM, the most risky sun-related activity for adults is currently thought to be the fortnight's holiday with intense sunlight exposure. The heterogeneity in MSM influences the global photoreactivity of the skin. Although melanin-enriched MSM spots protect locally epidermal cells from UVL, the foci with lower melanin content are much less protected.

## 7. Conclusion

SCM development is under the influence of genetic factors, age, gender, and environmental influences. The present review was focused on the increasing incidence of SCM developed in women during their childbearing age. Many clinical aspects of the neoplasm are presently reported indistinguishable between genders. By contrast, the peritumoral skin exhibits some gender differences in the extent of physiologic MSM which appears less developed in women than in men. This would suggest that the field effect of prominent sun exposures is not primarily involved in the increasing incidence of SCM in young Caucasian women. These neoplasms generally correspond to thin SCM with a restricted germinative compartment corresponding to a neoplasm exhibiting a slow growth pattern.

The acquired discrete uneven skin pigmentation forming the MSM patterns is a hallmark of photoaging. Once delivered by melanocytes to keratinocytes, melanin acts in part as a UVL-filter. However, according to individual parameters including the phototype, age, and previous cumulative UVL exposures, skin presents distinct FMM appearances. The MSM pattern in men with CMM appeared more heterogeneous with a majority of them showing a large MSM extent.
